# Effect of centralization on geographic accessibility of maternity hospitals in Finland

**DOI:** 10.1186/s12913-020-05222-5

**Published:** 2020-04-21

**Authors:** Tiina Huotari, Jarmo Rusanen, Timo Keistinen, Tero Lähderanta, Leena Ruha, Mikko J. Sillanpää, Harri Antikainen

**Affiliations:** 1 Geography Research Unit, University of Oulu, PO Box 3000, FI-90014 University of Oulu, Oulu, Finland; 2 Ministry of Social Affairs and Health, Finland, PO Box 33, FI-00023 Government Helsinki, Finland; 3 Research Unit of Mathematical Sciences, University of Oulu, PO Box 3000, FI-90014 University of Oulu, Oulu, Finland

**Keywords:** Accessibility, Geographic information system, Health care reform, Maternity hospitals, *p*-median, Capacitated location-allocation

## Abstract

**Background:**

In the past two decades, the number of maternity hospitals in Finland has been reduced from 42 to 22. Notwithstanding the benefits of centralization for larger units in terms of increased safety, the closures will inevitably impair geographical accessibility of services.

**Methods:**

This study aimed to employ a set of location-allocation methods to assess the potential impact on accessibility, should the number of maternity hospitals be reduced from 22 to 16. Accurate population grid data combined with road network and hospital facilities data is analyzed with three different location-allocation methods: straight, sequential and capacitated *p*-median.

**Results:**

Depending on the method used to assess the impact of further reduction in the number of maternity hospitals, 0.6 to 2.7% of mothers would have more than a two-hour travel time to the nearest maternity hospital, while the corresponding figure is 0.5 in the current situation. The analyses highlight the areas where the number of births is low, but a maternity hospital is still important in terms of accessibility, and the areas where even one unit would be enough to take care of a considerable volume of births.

**Conclusions:**

Even if the reduction in the number of hospitals might not drastically harm accessibility at the level of the entire population, considerable changes in accessibility can occur for clients living close to a maternity hospital facing closure. As different location-allocation analyses can result in different configurations of hospitals, decision-makers should be aware of their differences to ensure adequate accessibility for clients, especially in remote, sparsely populated areas.

## Background

Finland is a sparsely populated country where efficient and equal provision of health care services is challenging. Declining birth rate, together with the need to control public spending on health care, have led authorities to consider the extent at which maternity health services are provided in the country [1]. The closure of several maternity hospitals already enforced in the past few years as part of the centralization effort has raised concern in the affected regions. The debate is centered around the question of adequate accessibility, that is, how quickly and safely can the expectant clients reach a maternity hospital. Finland’s Constitution [2] states that public authorities must ensure, according to the law further provided, adequate social and health care services, and promote the health of the population. For this reason, there is a need to evaluate how closures of maternity hospitals would affect accessibility, and to ensure that the implications of political decisions are equitable in terms of accessibility.

**Fig. 1:**
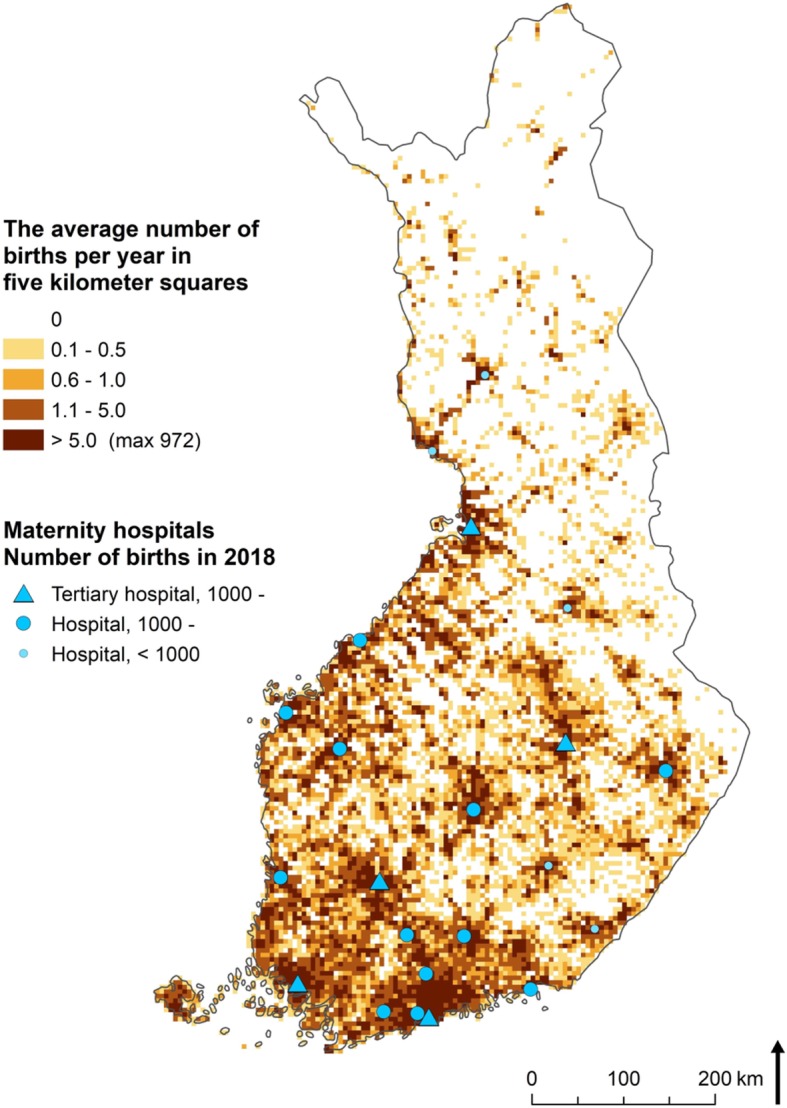
Maternity hospitals in 2019 and the estimated average number of children born per year in five-kilometer grid cells. Map data derived from the Monitoring System of Spatial Structure and Urban Form (YKR), provided by Statistics Finland and the Finnish Environment Institute

In Finland, public health care is in charge of maternity services, currently provided in 22 hospitals in mainland Finland (excluding the Åland islands with an extensive self-government) (Fig. 1). Five of these hospitals are tertiary-level university hospitals, while the rest are level 2 hospitals. Although most parts of Finland are inhabited, most of the population of 5.5 million people is concentrated in the regional capital cities and the southern part of the country. Eastern and northern parts of the country are predominantly rural. Even if hospital accessibility in Finland is mostly good, there are areas in northern Finland where the travel time to the nearest hospital is several hours by road transport. Almost two thirds of the maternity hospitals in Finland have been shut down over the last decades: in 1975, there were as many as 62 maternity units, while 42 units still remained in 1999 [3]. At the time of writing this article, the number of maternity hospitals had just been reduced to 22.

Although a temporal correlation has been discovered between an increase in the accidental out-of-hospital birth rates (births on the way to the hospital or unplanned at home) and closing of small hospitals in Finland [4], maternity health care is generally considered to be of high quality in Finland. For example, perinatal mortality (stillbirths and deaths before 7 days) per 1000 live births was 3.9 in year 2017 [5]. Maternal mortality is also low: in 2014 there were three maternal deaths, which is the same as the ten-year average 2005–2014, corresponding to maternal mortality of 5.2 deaths per 100,000 live-born children [6]. A basic requirement for a maternity hospital in Finland is to have a gynecological specialist, anesthetist and enough staff for the operation theatre in case of emergency Caesarean section [7]. Almost all labors (99.5%) take place in maternity hospitals, but the share of babies born outside of hospital either unplanned or while on the way to the maternity hospital was 0.37% in 2016 and 0.36% in 2017 [5].

Accessibility analyses have been increasingly utilized in health care studies in the past few decades. This is partly due to a proliferation of software tools and geographical data sources enabling the assessment of accessibility and the match between the demand and supply of health care. Most of these studies have focused on the accessibility conditions of a certain region or accessibility of a certain type of health care service. The regional differences in the accessibility of maternity services have been studied in Australia [8], Niger [9], and China [10], all of which are geographically extensive countries. Some studies have also been devoted to the risk factors related to the accessibility of maternity hospitals and services. In a study concerning planned hospital deliveries in the Netherlands, Ravelli et al. [11] found that a 20-min travel time by car from one’s home to the hospital is associated with an increased risk of mortality and adverse outcomes. Similarly, in two studies conducted in France, travel time thresholds of 30 min [12] and 45 min [13] were associated with an increased risk of accidental out-of-hospital deliveries and adverse perinatal outcomes. Further, a study in remote, rural areas in Canada concerning threshold travel times found that travel times longer than one hour increased the chance of complications and the perinatal mortality was highest in communities located more than 4 h from maternity services [14].

However, while good accessibility of maternity services is apparently related to decreased risk of adverse outcomes, there are several other factors which also contribute to safety. One such thing is client volume and the associated expertise of personnel. For example, emergency Caesarean section is needed in approximately 1% of deliveries in Finland [5]. Continuous preparedness, also during off-office hours, is costly especially in the units with a low number of deliveries per year. This leads to a high per delivery cost and political pressure to reduce the number of maternity hospitals. Centralization is, therefore, generally viewed as one of the approaches that is suited to improving quality of care [15]. According to a study carried out in Norway, births should be centralized to bigger units, where the number of neonatal deaths is significantly lower than in smaller units [16]. It has also been discovered that the risk of early-neonatal death in low-risk births in smaller delivery units (less than 500 births yearly) is substantially increased when compared with low-risk births in larger units (more than 1500 births yearly) [17]. In Finland, a thousand births has been suggested as a rough guideline for a hospital’s minimum annual volume. If meticulously enforced, this criterion would entail the sacrifice of accessibility in the remote and sparsely populated parts of the country. The number and configuration of maternity hospitals is, inevitably, a trade-off between safety aspects related to a facility itself, and that of travel time. Nevertheless, the centralization of births into larger units results in larger catchment areas and decreased accessibility in some areas, making it important to understand the difficulty associated with organizing maternity hospitals to ensure a unit’s sufficient size and safety and, on the other hand, adequate accessibility even in remote areas.

### Aim of the study

The aim of our study is to evaluate the impact that a hypothetical reduction of maternity hospitals from the currently operating 22 hospitals to 16 hospitals might have on accessibility in mainland Finland. Although there are many opinions regarding what an adequate number of maternity hospitals in Finland is, and no generally agreed number exists, 16 is the lowest possible number that has been suggested by authorities so far. We acknowledge that the justification for choosing this particular figure is vague, but it is supported by the fact that currently, in six hospitals the annual number of births is either below the recommended minimum level of one thousand, or only very slightly above it.

In 2010, when 30 maternity hospitals were still operating in mainland Finland, up to almost 79 and 96% of deliveries were associated with a less than 30 min and an hour travel time to the nearest maternity hospital, respectively [18]. As the number of hospitals continued to decrease throughout the 2010s, many smaller cities were left without their own hospital, increasing travel times. Although the closures may not initially have a major impact on accessibility, the continuing closures may eventually cause large regions to become underserved, as distances between remaining hospitals increase. As a result, further reductions have the potential to drastically deteriorate accessibility, justifying the need for conducting an analysis in advance. This is also important because closures can be realized based on different grounds. Most typically, the smallest units in terms of the number of births or units in the vicinity of another large unit are the ones which will be closed first. However, this may not always be the best option when accessibility is concerned.

In this study, we compare four alternative scenarios for reducing the number of maternity hospitals from 22 to 16. Starting from the most straightforward scenario of simply retaining the 16 hospitals currently having the highest volumes of demand and transferring the demand of closed hospitals to the nearest remaining hospital, we delve more deeply into three different ways of using location-allocation for determining the best configuration of hospitals in terms of accessibility. First, we use location-allocation to directly determine the set of 16 hospitals minimizing the total travel time. Secondly, we perform location-allocation sequentially, in which one hospital with the lowest volume is dropped from the configuration, and its demand is allocated to the remaining hospitals. The process is repeated until 16 hospitals are left. We also implement a distance minimization approach with capacity constraints for the hospitals to ensure that none of them will have too little or too much demand allocated to it. This type of restriction is important in real-life scenarios where hospitals have limits to the volume of demand they can handle, or the minimum level of demand they are required to have.

Closure of any health care service facility is likely to create a gap in accessibility and therefore, its effects should be known before any actions are taken. We expect our analyses to provide insights into the decision-making process concerning the provision of maternity services especially in terms of accessibility and regional differences in the level of service provision.

## Methods

Generally, three kinds of data are needed in the analysis of service accessibility [19]*.* First, information about the spatial distribution of the service users is needed. In this study, we utilized grid-based demographic statistics from the last day of year 2018 provided by Statistics Finland, because small areal units of regular size are most appropriate for accessibility analyses based on a transport network. Although the spatial precision of the grid data is 250 m × 250 m, we aggregated the data into larger five-kilometer grid squares in order to facilitate computations. Because of the large age range of fertile women, we used the population of 0–6-year-old children to represent areas where mothers, the users of maternity hospitals, live. Secondly, the locations of service facilities are required. In this study, we obtained the addresses of the maternity hospitals and geocoded them into x/y coordinates. Finally, we used the Digiroad database, a national road and street database maintained by the Finnish Transport Agency [20], to estimate travel times between facilities and their clients. In this study, we consider travel time by car only. We estimated the travel times according to the maximum allowed driving speed, while taking the slower speeds on the more congested roads in cities into consideration, as well as adding a time penalty for any right or left turns.

We performed the uncapacitated location-allocation analyses with the Network Analyst extension of ArcGIS software (Esri, Redlands, CA). Specifically, the location-allocation problem was solved as a *p*-median problem which, in general terms, requires siting *p* facilities among a set of *m* candidate sites to serve *n* (weighted) demand sites, so that the overall total travel cost is minimized [21]. As stated above, we applied the *p*-median analysis in three different ways. First, we performed the analysis to directly find the 16 hospitals which minimize the overall distance traveled. However, we also performed a sequential analysis in which one hospital is dropped according to the number of births, but at each step, a reallocation of demand is performed for the remaining hospitals. In other words, the smallest one, based on allocated demand, was first removed and demand was reallocated to the remaining hospitals using the *p*-median method. We then repeated the process until 16 hospitals were left. This procedure can simulate real-world decision-making processes, in which the number of hospitals is reduced gradually instead of a major reduction involving the closure of several facilities at the same time. As a third approach, we used a capacitated *p*-median method which, in addition to minimizing the distance between clients and hospitals, can also restrict the hospital size by setting a lower (1000) and an upper (9000) limit for the number of clients in each hospital. The upper limit we used is a crude estimate of capacity determined according to the highest annual number of deliveries in any of the currently operating hospitals (8966), whereas the lower limit is based on proposed safety guidelines. Since these types of restrictions are not implemented in ArcGIS, we used the rpack function in the R-package instead [22, 23]. We refer to the three different location-allocation analyses as ‘straight *p*-median’, ‘sequential *p*-median’ and ‘capacitated *p*-median’.

Loosely based on critical travel time thresholds identified in previous studies, we selected the travel times of 30 min, one hour and two hours as three alternative time limits, and we also used these thresholds to classify our results of demand coverage to four categories of accessibility labeled in the following way: excellent (< 0.5 h), good (< 1 h), moderate (< 2 h) and poor (> = 2 h). In order to facilitate evaluation, we calculated a reference value indicating the best demand coverage that any configuration of 16 hospitals can provide within each time limit. This was performed with ArcGIS by means of a maximal coverage analysis [24], which maximizes the volume of demand within a given distance threshold. Since we defined three time limits, the analysis was carried out three times, once for each time limit. As the outcome of maximal coverage analysis is optimized separately for each threshold, the solutions may be different across time limits. Maximal coverage analysis cannot be used for actual optimization purposes unless a definite time or distance threshold for accessing a service exists; however, it can be used for determining benchmark values, as we do in this study.

## Results

**Fig. 2:**
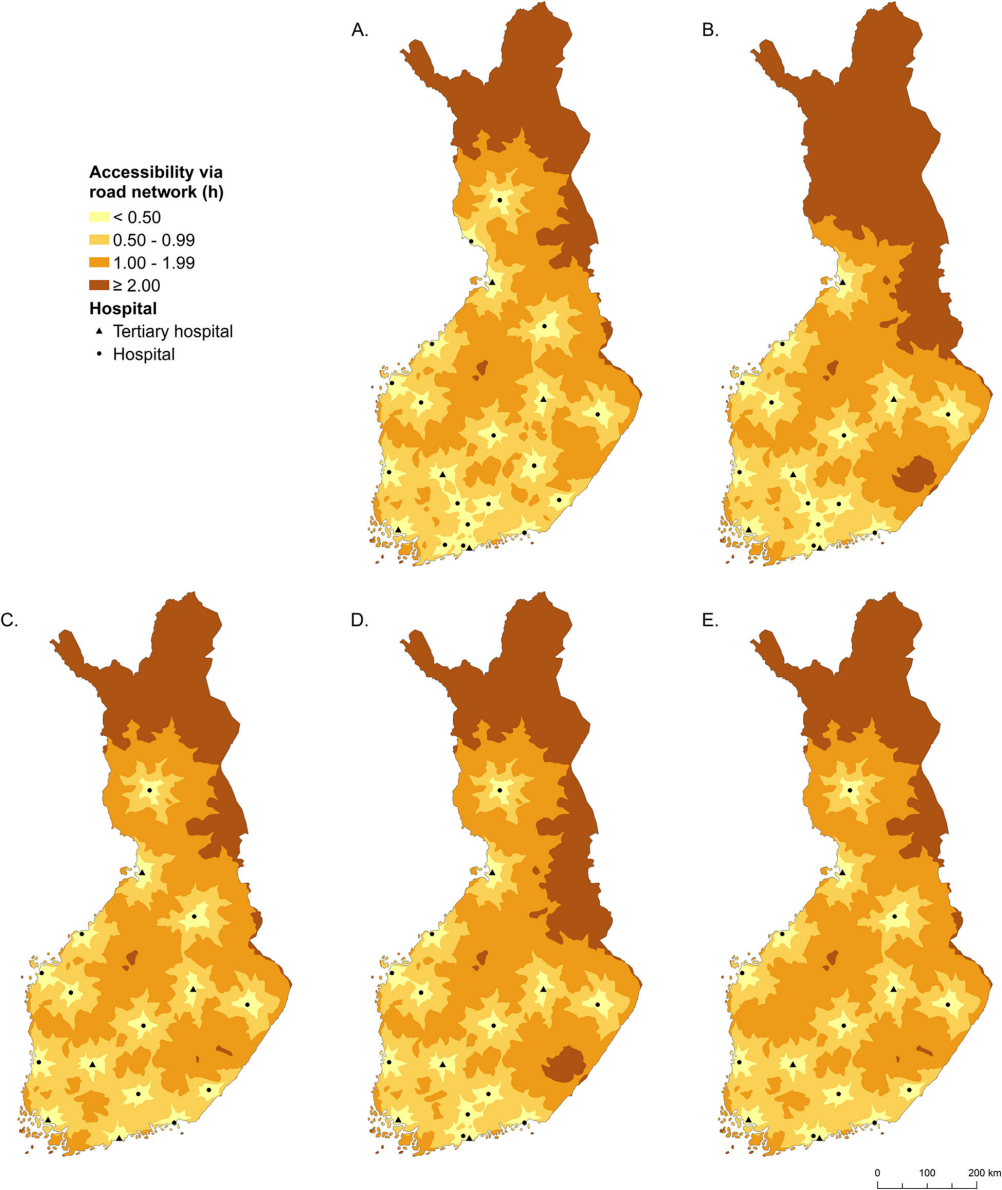
Accessibility of maternity hospitals and travel time zones in Finland. **a** In the current situation of 22 units, **b** the 16 largest units, **c** 16 units selected using straight *p*-median analysis, **d** 16 units selected using sequential *p*-median analysis, and **e **16 units selected using capacitated *p*-median analysis

In the current situation of 22 maternity hospitals in mainland Finland, overall accessibility can be considered to be good (Table 1, Fig. 2). Almost 70% of demand is located within less than 30 min travel from the nearest maternity hospital and only 0.5% of demand is located more than two hours away from the nearest hospital. The poorest accessibility conditions can be found in the remote areas in the north of the country, whereas in the south, some hospitals are located only about 30 min apart in terms of travel time by car. Note that as a result of the use of hypothetical average values for birth volumes and the allocation of demand to the nearest hospital, the maximum volume of demand allocated to a hospital (10488) is somewhat higher than the actual maximum number of births in a single hospital (8966) recorded in 2018.

**Table 1:** The percentage of demand divided into zones of accessibility according to travel time

Accessibility level (travel time in hours)	Current situation (22 hospitals)	16 largest hospitals	Location-allocation analysis			Best possible coverage ^a^
			Straight *p*-median	Sequential *p*-median	Capacitated *p*-median	
Excellent (< 0.5)	69.4	63.7	61.1	63.6	60.4	63.9
Good (< 1)	93.1	86.4	89.6	87.5	88.4	90.0
Moderate (< 2)	99.5	97.3	99.4	99.0	99.4	99.5
Poor (≥ 2)	0.5	2.7	0.6	1.1	0.6	–
Mean	26 min 23 s	33 min 35 s	30 min 23 s	31 min 6 s	30 min 50 s	
Capacity of hospital						
Maximum	10488	10488	17515	10488	8999	
Minimum	582	1126	1056	1419	1056	

The travel time is calculated for the current situation and four different scenarios involving only 16 maternity hospitals

^a^Best possible coverage is calculated separately for each travel time threshold using maximal coverage analysis with 16 maternity hospitals

In a hypothetical situation where all births were centralized into the 16 maternity hospitals, which are the largest according to birth volumes resulting from assigning demand always to the nearest hospital, the share of clients in the two best accessibility categories would fall significantly compared to the presence of 22 hospitals. Even if this scenario satisfies the minimum requirement of a thousand births a year in every remaining hospital, and the share of demand located in the best accessibility category is only marginally worse than the reference value, the percentage share of the poorest accessibility category increases by more than five-fold compared to the current situation. The reason for the marked deterioration of accessibility in this scenario is the closure of two small but regionally important hospitals in the north of Finland (Fig. 2). Hence, the scenario may not be realistic due to the marked regional disparities in accessibility.

While it is obvious that reducing the number of maternity hospital facilities inevitably impairs accessibility in any scenario, the rationale for employing location-allocation analyses is the possibility to achieve a more geographically balanced configuration of hospitals. Straight, uncapacitated *p*-median analysis is generally the most commonly used type of location-allocation analysis, and it would likely be the first-choice method for facility site selection in the real world. It is interesting to note that by using this approach to cut down the number of hospitals from 22 to 16, the volume of demand falling in the ‘poor’ accessibility category would hardly change at all from the current situation. Also, in this scenario, the volume of the smallest hospital would be above the critical level of one thousand, without enforcing a lower limit in the allocation. However, while accessibility would mostly remain ‘moderate’ in this case, the closures would result in a considerable shift from short distances to distances longer than one hour. The foremost problem with this scenario is, however, the concentration of almost a third of all demand is directed to a single hospital. This is because only one hospital would be retained in the densely populated capital city region of Finland. In such a situation, problems in hospital logistics would be likely, possibly negating the benefits of centralization.

The scenario related to the sequential *p*-median analysis, aiming to mimic real-world decision-making in which hospitals are closed one by one in the order determined by their client volumes, results in a higher proportion of demand to be placed in the poorest accessibility category than in the straight *p*-median analysis. Interestingly, this also results in a higher percentage of demand in the best accessibility category. The result is similar to that of retaining the 16 largest hospitals, giving rise to regional disparities in accessibility. However, the method also has an interesting capability of producing a more balanced solution in terms of the distribution of demand across hospitals. The lowest allocated demand is the highest of all scenarios assessed here, which can be considered a desirable result, while the maximum allocated demand is reasonably low. It appears that the way in which stepwise location-allocation reallocates the demand of a dropped facility to the remaining facilities is not only realistic in terms of real-world planning, it also provides a regionally balanced solution.

The capacitated version of the *p*-median produces quite similar results as the previous two methods, however this scenario has the lowest proportion of demand in the best accessibility category. On the other hand, by setting the capacity constraints to [1000, 9000], we ensure a more balanced allocation of demand and that the number of births in each hospital per year is reasonable. Enforcement of capacity limits inevitably has a negative impact on accessibility, but this seems to be limited to the best accessibility category. In other accessibility categories (‘good’ and ‘moderate’ accessibility) the negative impact is far less apparent and, in fact, for these categories the solution is superior to that of sequential *p*-median analysis, and reasonably close to the reference values. As only 0.6% of demand is located in the worst accessibility category, the enforcement of capacity limits can be regarded as a regionally justifiable approach as far as regional equality is concerned.

## Discussion

In this study, we evaluated a set of alternative configurations for maternity hospitals in Finland in a hypothetical situation where the number of maternity hospitals would be 16 instead of the current number of 22. Accessibility is inevitably affected as a result of closures: even in the current situation, the proportion of excellent accessibility, for instance, has fallen by about ten percentage units compared to a situation a decade ago. However, the outcome is different depending on the scenario used to select remaining hospitals. While the scenario seeking to centralize services into the highest-volume units results in excellent accessibility for the largest share of clients, it is also associated with regional disparities in accessibility. On the other hand, scenarios based on location-allocation, oriented towards securing the best overall accessibility of hospitals, provide better results for the categories of ‘good’ (travel time less than one hour) or ‘moderate’ (travel time less than two hours) accessibility.

Although the differences between the results related to the different scenarios may appear quite small, it is noteworthy how hospitals in the largest population centers are almost always included in the configuration regardless of the used analysis method. As a result, accessibility conditions remain unchanged for the majority of clients, whereas drastic changes can occur for a minority of clients without this being clearly noticeable in the overall outcome. In the case of Finland, this problem concerns, in particular, the low-volume hospitals in the north. It is also important to realize that differences in accessibility can be underestimated due to the possibility of masked changes. The accessibility categories reveal only those shifts which occur between the categories, not the ones taking place inside the categories. For example, two different configurations of hospitals may seemingly provide an equal degree of ‘moderate’ accessibility, while distances can still be very different between these two configurations. In order to evaluate the possible presence of the masking effect in the results, we calculated the average change in travel time for the grid squares whose travel time was affected in the straight *p*-median analysis and capacitated *p*-median analysis, but which remained in the same accessibility category after reducing the number of hospitals from 22 to 16. Overall, the average change in travel time among these grid cells was less than nine minutes. A majority of changes occurred within the ‘excellent’ and ‘moderate’ categories, where the average change was less than eight minutes. For grid squares associated with a travel time longer than one hour, the average change was more pronounced, about 21 min, but the number of affected squares and their combined volume of demand was very low. We therefore conclude that masking is generally not a concern and changes in accessibility can be evaluated on the basis of shifts taking place across critical time thresholds. However, we acknowledge that masking may more severely affect results concerning areas with long travel times to the hospital.

### Policy considerations

This study was motivated by the need to evaluate the potential impacts on accessibility and service provision resulting from the closure of maternity hospitals. No assessment of accessibility was conducted preceding the closure of 12 maternity hospitals in Finland between 1999 and 2011. In the following years, aspects related to geographical accessibility started to attract more interest and tentative experiments with accessibility analyses were carried out, but their significance remained limited [25]. Indeed, the implications of accessibility reforms were largely overlooked in Finland until political decisions were made by the Finnish Government in 2017 concerning hospital services as well as many other public services. In some cases, the outcome of past reforms may have been reasonable and functional in terms of accessibility. The implications of closures can remain limited in the early stages due to the high initial density of hospitals, but as the closures proceed, the negative impacts on accessibility may become increasingly pronounced. In order to determine a turning point in which the closures are likely to trigger a reaction from clients, a dedicated analysis of accessibility is needed in advance.

Again, we want to stress that while accessibility of maternity hospitals can be seen as an utmost important aspect in decision-making concerning the reduction of these services facilities, it is by no means the sole relevant factor. The minimum limit of a thousand labors per year in a maternity hospital, suggested in some official reports in Finland, has been justified by the need to maintain the level of professional knowledge and technical ability to perform procedures, as well as limiting costs incurred due to the need for 24-h preparedness. The closure of one hospital will inevitably affect the client volume of one or more adjacent hospitals. If decisions regarding closures are made in every region separately, a balance of services is harder to achieve. There are highly valid reasons for retaining low-volume hospitals also in remote, sparsely populated areas. High-risk births, very preterm deliveries below 32 gestational weeks and neonates with problems requiring surgery are already centralized to the five tertiary hospitals in Finland. As the referral system for handling high-risk births in Finland is established and well-functioning [26], the added benefit of centralizing maternity services to high-volume facilities, regardless of accessibility, may not be enough to outweigh the increased risk incurred by longer travel times. Nevertheless, recent research shows that centralization has been followed by an improvement in very preterm infants’ survival [27], suggesting that a critical mass of expertise is highly important in ensuring high-quality care.

### Limitations

Aside from the fact that this study was designed to focus solely on geographic accessibility, thus excluding many other considerations affecting the provision of maternity hospital services, we acknowledge there are several limitations involved in analyses performed in the study.

First, while the gridded population data provide a regular decomposition of space which is better suited to location-allocation than administrative units of irregular shapes and sizes, they do not include information about the geographic distribution of expectant mothers. In this study, the analyses are based on the assumption that expectant mothers are living in the same areas as children from newborn up to 6 years. We have calculated the average births from this seven-year data set in every grid square, and the average number of children born in a year represents mothers who are using maternity hospital services. We presumed this estimate to indicate the true location of births better compared to, for example, all women of fertile age. An added benefit of this is that the results are directly applicable to hospital services for children as well.

As another study limitation, accessibility of maternity hospitals was determined only by car transport. However, since car is an overwhelmingly dominant mode of transport in Finland, it is reasonable to restrict the analyses to this mode of transport. In addition, because it is often impossible to predict the moment of birth accurately, one must rely on a passenger vehicle or taxi instead of using public transport.

## Conclusions

Even if the importance of service accessibility has been generally understood, it has not been widely taken into consideration in organizing health services in Finland. A possible explanation for this is that aspects related to accessibility have only become important as a result of the centralization of health services. Accessibility has not been a major concern in the era of expanding health services, or when closures have only affected small, local hospitals or clinics. Nowadays all maternity hospitals in Finland can be considered to be at least level 2 hospitals, and the closure of any of these hospitals will leave even relatively large cities along with their catchment areas without their own hospital.

The geographically scattered population structure in Finland requires some low-volume maternity hospitals to be retained especially in remote areas the north, despite centralization efforts. Nevertheless, a country-wide analysis of accessibility concerning the hospital network and potential clients in their entirety can be advocated as a fundamental tool for decision-making. Further, this study demonstrates that while the site selection for hospitals based on geographical accessibility may intuitively seem a straightforward task, the analyses can be carried out in different ways, resulting in different configurations and regional equality. In particular, the incorporation of capacity constraints into location-allocation analyses, which is often neglected due to methodological and computational challenges, can be considered an important contribution to geographical analysis of health care accessibility.

Although this study was geographically limited to a single country characterized by relatively long travel distances, extensive rural areas and a small and unevenly distributed population, the methods presented here are applicable to any countries where deliveries occur predominantly in hospitals. The implementation of the methods can also provide general insights into aspects of ongoing or planned centralization of health care services in settings where some hospitals are struggling to the meet the minimum volume standards regarding the number of clients or medical procedures.

## Data Availability

The data that support the findings of this study are available from Esri Finland and Statistics Finland, but restrictions apply to the availability of these data, which were used under license for the current study, and so are not publicly available. Data are however available from the authors upon reasonable request and with permission of Esri Finland and Statistics Finland.

## Abbreviations

GIS: Geographic Information System
YKR: Monitoring System of Spatial Structure and Urban Form

## References

[1] Ministry of Social Affairs and Health. Health and social services reform will be carried out focusing on services, reforming the structures simultaneously. 2019. https://stm.fi/en/artikkeli/-/asset_publisher/sote-uudistus-tehdaanpalvelut-edella-rakenteet-uudistetaan-samalla. Accessed 7 Jan 2020.

[2] Finland’s Constitution, 731/1999; 19§. http://www.finlex.fi/fi/laki/ajantasa/1999/19990731. Unofficial translation (Ministry of Justice, Finland) 2019. http://www.finlex.fi/en/laki/kaannokset/1999/en19990731.pdf. Accessed 11 Nov 2019.

[3] Tapper A-M (2011). Synnytyspalveluiden valtakunnallinen toteuttaminen. Selvityshenkilön raportti Sosiaali- ja terveysministeriö.

[4] Viisainen K, Gissler M, Hartikainen AL, Hemminki E (1999). Accidental out-of-hospital births in Finland: incidence and geographical distribution 1963-1995. Acta Obstet Gynecol Scand.

[5] Finnish Institute for Health and Welfare. Perinataalitilasto – synnyttäjät, synnytykset ja vastasyntyneet 2017. 38/2018. http://urn.fi/URN:NBN:fi-fe2018103146930.

[6] Official Statistics of Finland (OSF): Causes of death [e-publication]. ISSN=1799–5078. 2014, 7. Three maternal deaths in 2014. Helsinki: Statistics Finland 2014. http://www.stat.fi/til/ksyyt/2014/ksyyt_2014_2015-12-30_kat_007_en.html. Accessed 7 Mar 2020.

[7] Ministry of Social Affairs and Health. Yhtenäiset päivystyshoidon perusteet. Työryhmän raportti. Sosiaali ja terveysministeriön selvityksiä. 2010;4:102.http://urn.fi/URN:ISBN:978-952-00-2963-0.

[8] Longman J, Pilcher JM, Donoghue DA, Rolfe M, Kildea SV, Kruske S, Oats JJN, Morgan GG, Barclay LM (2014). Identifying maternity services in public hospitals in rural and remote Australia. Aust Health Rev.

[9] Blanford JI, Kumar S, Luo W, MacEachren AM (2012). It’s a long, long walk: accessibility to hospitals, maternity and integrated health centers in Niger. Int J Health Geogr.

[10] Song P, Zhu Y, Mao X, Li Q, An L. Assessing spatial accessibility to maternity units in Shenzhen, China. PLoS One. 2013;8(7):e70227. 10.1371/journal.pone.0070227. 10.1371/journal.pone.0070227 PMC3716609 23894622

[11] Ravelli AC, Jager KJ, de Groot MH, Erwich JJ, Rijninks-van Driel GC, Tromp M, Eskes M, Abu-Hanna A, Mol BW (2010). Travel time from home to hospital and adverse perinatal outcomes in women at term in the Netherlands. BJOG.

[12] Combier E, Charreire H, Le Vaillant M, Michaut F, Ferdynus C, Amat-Roze J-M, Gouyon J-B, Quantin C, Zeitlin J (2013). Perinatal health inequalities and accessibility of maternity services in a rural French region: closing maternity units in Burgundy. Health Place.

[13] Renesme L, Garlantézec R, Anouilh F, Bertschy F, Carpentier M, Sizun J (2013). Accidental out-of-hospital deliveries: a case–control study. Acta Paediatr.

[14] Grzybowski S, Fahey J, Lai B, Zhang S, Aelicks N, Leung BM, Stoll K, Attenborough R (2015). The safety of Canadian rural maternity services: a multi-jurisdictional cohort analysis. BMC Health Ser Res.

[15] Tanke MAC, Ikkersheim DE (2012). A new approach to the tradeoff between quality and accessibility of health care. Health Policy.

[16] Moster D, Lie RT, Markestad T (2001). Neonatal mortality rates in communities with small maternity units compared with those having larger maternity units. BJOG..

[17] Heller G, Richardson DK, Schnell R, Misselwitz B, Künzel W, Schmidt S (2002). Are we regionalized enough? Early-neonatal deaths in low-risk births by the size of delivery units in Hesse, Germany 1990-1999. Int J Epidemiol.

[18] Huotari T, Antikainen H, Pukkinen M, Rusanen J (2012). Synnytyspäivystyksen ja erikoissairaanhoidon palveluiden saavutettavuus. Sairaaloiden sijainnin suhde väestörakenteeseen paikkatietomenetelmillä tarkasteltuna. Sosiaali- ja terveysministeriön raportteja ja muistioita.

[19] Tanser F, Gething P, Atkinson P. Location-allocation and Planning. 540–566. In the Book Brown T, McLafferty S, Moon G. 2010. A companion to health and medical geography. Blackwell Publishing Ltd. 610 p.

[20] Finnish Transport Agency. 2019. https://vayla.fi/web/en/open-data/digiroad. Accessed 18 Dec 2019.

[21] Miller HJ, Shaw S-L (2001). Geographic information systems for transportation: principles and applications.

[22] Lähderanta T, Leppänen T, Ruha L, Lovén L, Harjula E, Ylianttila M, Riekki J, Sillanpää MJ. Edge computing server placement with capacitated location allocation. arXiv preprint. 2020. https://arxiv.org/pdf/1907.07349.pdf.

[23] Lähderanta T, Lovén L, Ruha L (2019). rpack: R package for capacitated clustering.

[24] Church R, ReVelle C (1974). The maximal covering location problem. Pap Reg Sci.

[25] Huotari T, Antikainen H, Keistinen T, Rusanen J (2017). Accessibility of tertiary hospitals in Finland: a comparison of administrative and normative catchment areas. Soc Sci Med.

[26] Hemminki E, Heino A, Gissler M (2011). Should births be centralised in higher level hospitals? Experiences from regionalised health care in Finland. BJOG.

[27] Helenius K, Gissler M, Lehtonen L (2019). Trends in centralization of very preterm deliveries and neonatal survival in Finland in 1987–2017. Translational Pediatrics.

